# Characterization of some physicochemical, textural, and antioxidant properties of muffins fortified with hydrolyzed whey protein

**DOI:** 10.1002/fsn3.4422

**Published:** 2024-08-20

**Authors:** Hatice Bekiroglu, Safa Karaman, Fatih Bozkurt, Osman Sagdic

**Affiliations:** ^1^ Department of Food Engineering, Faculty of Agriculture Sirnak University Sirnak Turkey; ^2^ Department of Food Engineering, Faculty of Chemical and Metallurgical Engineering Yildiz Technical University Istanbul Turkey; ^3^ Faculty of Engineering, Department of Food Engineering Nigde Omer Halis Demir University Nigde Turkey

**Keywords:** emulsion stability, hydrolysis degree, oxidative stability, thermal properties

## Abstract

Whey protein hydrolysates, derived from enzymatic hydrolysis of whey protein isolates or concentrates, offer enhanced bioavailability and solubility compared to intact whey protein. In this study, whey protein hydrolysates (WPHs) having different hydrolysis degrees (5%, 10%, and 15%) were produced and muffin cakes were enriched with the addition of WPHs. In general, the addition of WPHs showed a significant effect on oil and protein content while the emulsion activity was improved with the increased hydrolysis degree (HD). The degree of hydrolysis increment resulted in a significant increase in both antioxidant power and antiradical activity of the WPHs. Ferric‐reducing antioxidant power and ABTS radical scavenging activity ranged between 18.83–87.27 mg TE/100 g and 211.8–5063.1 mg TE/100 g, respectively. The highest FRAP and ABTS values were recorded for the 15% HD while the lowest was for the native whey protein isolate (WPI). The induction periods giving a clear information for the oxidative stability were 1593 min for the control muffins, and it was 1654 for the muffin added with WPI. Rheological data revealed that all cake batter samples including WPHs showed viscoelastic behavior. WPHs could be efficiently used in muffin formulation to increase the biofunctional effects of the final products.

## Graphical abstract

The study investigated the impact of whey protein hydrolysates (WPHs) with varying degrees of hydrolysis on muffin cakes. Muffin cake samples enriched with WPHs demonstrated notable antioxidant and antiradical properties. All batter samples containing hydrolyzed whey protein exhibited an elastic gel‐like texture and a soft gel structure.

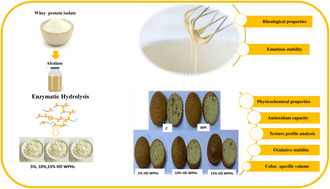

## 1 INTRODUCTION

Protein hydrolysates, also known as bioactive peptides, have become increasingly popular as natural biofunctional ingredients in recent years. They can be naturally occurring or produced using various hydrolysis techniques (Sinthusamran et al., 2019). Enzymatic hydrolysis is an environmentally friendly modification process that provides high yields of quality and purpose‐oriented bioactive peptides under optimized conditions and does not allow the formation of undesirable by‐products that occur in other hydrolysis techniques. The protein hydrolysates are known to have important biofunctional activities such as mineral binding activity, antimicrobial, antioxidant, immunomodulatory, and antihypertensive functions (Cumby et al., 2008; Shimizu et al., 2008). Functional peptides can play multiple roles in foods, affecting their texture, flavor, stability, and nutritional value. Understanding the interactions between these peptides and food matrices is essential for the development of innovative and improved food formulations. Both of these interactions can have various effects on the quality, texture, and shelf life of foods (Abd El‐Salam & El‐Shibiny, 2017; Sodini et al., 2005; Souppe & Prodhomme, 2006; Zhao et al., 2006). The degree of protein hydrolysis refers to the extent to which a protein has been broken down into smaller peptides and amino acids through hydrolysis. The HD of protein is an important factor in determining how protein‐derived ingredients and hydrolysates can be used in various food products.

Due to these positive health effects of the protein hydrolysates, commonly consumed food formulations are started to be fortified with the addition of bioactive compounds. For example, Mohammadi et al. (2022) studied the effect of incorporating lentil protein hydrolysates into muffins on its physicochemical and sensory attributes while Ambigaipalan and Shahidi (2015) investigated some characteristic properties of the muffin fortified with date seed flour and its hydrolysates. Research on the potential use of milk protein hydrolysates in bakery products and the techno‐functional characteristics that hydrolysates provide and enhance on a product basis appears to be somewhat limited. In one of these studies, Gani, Broadway, Ahmad, et al. (2015) examined the effect of casein and whey hydrolysates on the rheological, textural, and sensory properties of cookies, and determined that cookies with hydrolysates were more preferred in color and sensory liking tests. Gani, Broadway, Masoodi, et al., 2015 investigated the possibilities of using milk protein and hydrolysates in different amounts in bakery and reported that the 10%–15% protein hydrolysates added to the formulation significantly improved the nutritional quality.

Whey protein, a by‐product of the cheese‐making process is reported to be a highly nutritious, functional, and biologically active source of protein. It is quite suitable for the hydrolysis process because it was reported that the peptides obtained by the hydrolysis of the whey protein had strong antioxidant activity (Hernández‐Ledesma et al., 2005; Peńa‐Ramos & Xiong, 2001; Peña‐Ramos & Xiong, 2003). It was reported that the whey protein hydrolysates were more effective in terms of radical scavenging performance compared to whey protein (Dryáková et al., 2010) because during the hydrolysis process of proteins, the tertiary structure of the protein is disrupted and the solvent accessibility of released amino acids increases (Belobrajdic et al., 2003). Mohan et al. (2015) noted that even a low degree of hydrolysis of whey proteins causes significant structural changes, allowing the production of hydrolysates with improved antioxidant activity. This strong radical binding capacity released by hydrolysis enables the use of whey‐based substrates as antioxidant carriers in a wide range of dairy and confectionery products (Embiriekah et al., 2018). Kumari et al. (2013) observed that 1% flavor and alcalase enzyme hydrolysates of whey protein added to chocolate ice cream caused an increase in antioxidant activity up to 14.73% and 29.6%. In another study, whey and its hydrolysates were utilized to enrich strawberry and chocolate‐flavored milk, and it was reported that the hydrolysates provided an even higher antioxidant capacity of up to 42% compared to control and isolate‐containing samples (Mann et al., 2015). In addition, whey protein and its hydrolysates‐based ingredients are frequently used in a wide range of products to take advantage of their nutritional and functional properties, such as their high emulsifying capacity (de Figueiredo Furtado et al., 2021; Li et al., 2023). It requires additives used in heterogeneous systems such as cakes to be much more functional in terms of providing the desired product‐specific structural characters and the formation of strong emulsions at oil–water interfaces.

To the best of our knowledge, no studies have examined the effects of using the same protein source at different hydrolysis degrees on the technological and functional characteristics of products, such as cakes. Despite studies investigating the use of protein hydrolysates in various foodstuffs and their resulting structural changes (Brown et al., 2023; Cermeño et al., 2021; Mohammadi et al., 2022), there is a gap in research when it comes to understanding how these factors affect muffin cakes.

It is essential to enhance the antioxidant capacity of products such as muffin cakes, which are consumed by people of all ages, especially children, by using whey hydrolysate to improve the quality of functional foods. Additionally, this study holds significance in evaluating the contribution of hydrolysates with varying degrees of hydrolysis to structural properties such as volume increase, hardness, and other factors that influence consumer preferences for muffin cakes.

The main scenario behind this work was to reveal the effect of HD on some physicochemical, textural, and antioxidant properties of whey protein hydrolysates added to muffin cake samples.

## 2 MATERIALS AND METHODS

### 2.1 Materials

For the muffin cake production, egg, table sugar, vegetable oil, whole milk (Sütaş, Türkiye, wheat flour, baking powder), vanilla (Dr. Oetker, İzmir, Türkiye), and shortening (Ülker Bizim, Besler, Türkiye) were purchased from local markets in Istanbul, Türkiye. Whey protein isolate (WPI) (96 g protein/100 g powder) was provided by Hard Line Nutrition. Alcalase (protease from *Aspergillus oryzae*), 2,2‐diphenyl‐1‐picrylhydrazyl (DPPH), 2,4,6‐ tris(2‐pyridyl)‐*s*‐triazine (TPTZ), 2,2′‐azinobis‐3‐ethylbenzothiazoline‐6‐sulfonic acid (ABTS), and all the other chemicals used were purchased from Sigma‐Aldrich (St. Louis, MO, USA).

### 2.2 Methods

#### 2.2.1 Whey protein hydrolyzation process

The alcalase enzyme was used to produce WPHs having different degrees of hydrolysis. Briefly, 10 g of WPI was mixed with distilled water (5:100; protein: water) and dissolved thoroughly using a vortex mixer (Isolab, Germany). For the hydrolysis process, the whey dispersion's pH was adjusted to 8 and the temperature was set to 60°C as the optimum working conditions for the alcalase enzyme. The enzyme was added to the whey dispersions with a 1:99 enzyme‐substrate ratio (v/v). As stated in the previous study (Bekiroglu et al., 2022), hydrolysis was carried out according to the required 0.1 N NaOH amount to obtain whey hydrolysates with a HD of 5%, 10%, and 15% calculated by the pH‐stat method (Adler‐Nissen, 1986). For the determination of HD of the peptides, the ratio of the peptide bonds (h) broken as a result of hydrolysis to the total number of bonds per unit weight (htot) was determined and the degree of hydrolysis was calculated according to the following Equation (1):

(1) $$\%\mathrm{DH}=h\times 100/\text{htot}=\left(B\times N_{b}\times 100\right)/\left(\alpha \times M_{p}\times \text{htot}\right)$$

where *B*, *N*_b_, α, and *M*_p_ are base amount, base normality, mean dissociation constant of the α‐NH_2_ groups, and the amount of protein (g), respectively. The total peptide bonds refer as htot (meqv/g protein) = 8.41 mmol/g protein (Zhang et al., 2012).

Immediately after the hydrolysis process was completed, the hydrolysate solutions were kept in a 90°C water bath for 10 min and the enzyme was inactivated. It was centrifuged at 7500 rpm for 30 min and the supernatant fraction was lyophilized using a freeze dryer (Martin Christ, Beta 1–8 LSC plus, Osterode am Harz, Germany) at −55°C and 1 hPa for 48 h to produce powdered WPHs. The hydrolysates were kept in a desiccator at 4°C until muffin cake production and all other analyses were carried out.

#### 2.2.2 Muffin cake manufacturing

The muffin cake samples were manufactured according to the process reported by Shaabani et al. (2018). The ingredients and their ratios used for the preparation of the muffin cakes are tabulated in Table S1 (see supplementary file). First, the cake mixes were prepared by mixing the sugar, vanilla, and oil in a high‐speed mixer (KitchenAid, Benton Harbor, MI, USA) for 1 min. The whole egg was then added and mixed for another 2 min. At the end of the duration, wheat flour, baking powder, and milk were incorporated into the first mix. The muffin samples were enriched with the addition of WPI and its hydrolysates produced at different HD (5%, 10%, and 15%) by replacing some of the wheat flour with 5% w/w (Table S1). Finally, the cake batters were baked for 30 min at 180°C in an electrical oven (Maksan, Nevşehir, Türkiye). After baking, the muffin samples (Figure S1, see supplementary file) were subjected to a cooling process for 1 h and the corresponding analysis was conducted for the muffin cakes.

#### 2.2.3 Determination of physicochemical properties of muffin cakes

The moisture content of the muffin samples was determined according to the AACC method 44–15.02 (AACC, 1990; Quiles et al., 2018). The water activity analysis of muffin samples was performed using a water activity meter (Novasina, LabTouch‐aw, Switzerland). The protein content of the muffin samples was determined using the Kjeldahl technique using the Behr Kjeldahl unit (Unit‐S5, Ahlen, Germany) applying a conversion factor of 6.25 (AACC, 1990). The oil level of the muffin cakes was determined by the Soxhlet extraction system (E816, Buchi, Flawil, Switzerland). Color values of the samples were measured using a calibrated colorimeter (CR‐400 Chroma Meter, Konica Minolta, Japan), and the color values were recorded as L*, a*, and b* for both crust layer and crumb of the muffin samples. The specific volume of the muffin batters was characterized according to the method suggested by Ammar et al. (2021). For this aim, cake‐specific volume was calculated as the ratio between the cake volume (mL) and its weight (g).

#### 2.2.4 Texture profile analysis of muffin cakes

Texture profile analysis of the muffin samples was conducted using a TA‐XT Texture Analyzer (TA‐XT2 Plus‐Stable Microsystem, England) equipped with a P/36R cylindrical probe, HDP/90 platform, and 5 kg of weight load (Krupa‐Kozak et al., 2020). Texture profile analysis was performed with compression mode, by pretest speed of 1.0 mm s^−1^, test speed of 1.0 mm s^−1^, and posttest speed of 5.0 mm s^−1^. TPA analysis was performed on the uncut shell section of each muffin sample with a diameter of about 40 cm, with the upper part not separated first. Then, 1 cm of the crust part was cut and removed, and force was applied to the remaining (crumb) inner part. The force (N) required to compress the slices to a 30% strain level was determined using the time (displacement) versus force graph. The parameters were hardness (g), cohesiveness (g), gumminess (g), chewiness (g), and resilience.

#### 2.2.5 Rheological characterization of muffin cake batters

##### Steady shear rheological characterization

Steady shear rheological characterization of the cake batter samples was performed by a strain–stress‐controlled rheometer (Anton Paar MCR 302, Austria) using a parallel plate probe (PP 25) at 25°C with a 1‐mm gap interval. Steady shear analysis was conducted in the shear rate range of 0.1–100 s^−1^. The recorded results were fitted to the Oswald de Waale model (Equation 1) and the model parameters, namely, consistency coefficient (K, Pas^n^) and flow behavior index (*n*) values were calculated.

(1) $$\tau =K\gamma^{n}$$

Where *τ* is the shear stress, *γ* is the shear rate, *K* is the consistency coefficient, and *n* is the flow behavior index (Rao & Cooley, 1992; Yoo & Rao, 1996).

##### Oscillatory shear rheological characterization

Dynamic mechanical spectra of the muffin batters enriched with WPHs were characterized by a strain‐controlled rheometer (Antonpaar MCR 302, Graz, Austria). First, a linear viscoelastic region was determined for the samples by application of an amplitude sweep test between 0.1% and 100% strain values. In the linear viscoelastic range, at constant strain (0.1%), the frequency sweep test was started between 0.1 and 100 rad/s angular velocity. Storage modulus and loss modulus values were recorded during the frequency sweep. Using the following equations, the model constants (*K*′ and *K*″) and slopes (*n*' and *n*″) were calculated by fitting to the viscoelastic parameters (Rao & Cooley, 1992; Yoo & Rao, 1996).

(2) $$G^{\prime }=K^{\prime }\omega^{n\prime }$$

(3) $$G^{\prime \prime }=K^{\prime \prime }\omega^{n\prime \prime }$$

#### 2.2.6 Emulsion stability analysis of muffin batters

Determination of emulsion stability of cake batters was carried out according to the gravimetric method described by Turabi et al. (2008). Twenty grams of each freshly prepared cake batter was weighed into a tared falcon tube and centrifuged at 6000 rpm for 20 min at 25°C. Immediately after centrifugation, the oil separated from the cake batters was removed and the amount of remaining batters was measured. The ratio of the amount of oil separated from the batters to the initial weight of the batters was subtracted from unity and multiplied by 100. The emulsion stability of the muffin batters was expressed as a percentage of emulsion stability.

#### 2.2.7 Thermal properties of the muffin samples

Thermal characteristics of muffin samples were evaluated based on a differential calorimetry scanning (DSC) method with a TA Instrument Q100 (New Castle, USA). The analysis was carried out according to the method described by Kemski et al., 2022 with some modifications. Approximately 10 ± 2.0 mg of cake sample was placed in hermetically sealed aluminum pans and heated from 25°C to 300°C with an isothermal ramp at 10°C/min in an inert atmosphere. A hermetically sealed aluminum pan was used as a reference. All measurements were performed under a constant flow of nitrogen gas at 50 mL/min for cooling. The onset temperature (*T*_0_), peak temperature (*T*_p_), and change of enthalpy ΔH values were calculated, which represent the thermal changes specific to each muffin cake sample.

#### 2.2.8 Bioactive characteristics of the muffin cake samples

##### Preparation of the extracts

For the extraction of the muffin cake samples, 2 g of cake sample was mixed with 6 mL of distilled water and homogenized by an ultra‐turrax homogenizer (Ultra‐Turrax, Daihan, HG‐15D, Gang‐Won‐Do, South Korea) at 10000 rpm for 5 min. Then, the extracts were centrifuged at 7500 rpm for 10 min (Hettich‐Zentrifugen, German) and the supernatant fraction was used for antioxidant analyses. For the extraction of the whey protein isolates (200 mg/mL) and hydrolysates (50 mg/mL), the samples were prepared with distilled water. The mixtures were placed on a shaking water bath (30°C) and 1 h later, the sample tubes were centrifuged at 7500 rpm for 10 min. After centrifugation, the supernatant was filtered by a 0.45‐μm syringe filter and the extracts were used for further bioactive analysis.

##### DPPH radical scavenging activity

DPPH radical scavenging activity of muffin cake extracts was determined according to the method suggested by Brand‐Williams et al. (1995). For this purpose, 100 μL of sample extract was mixed with 4.9 mL of DPPH solution in methanol (6 × 10^−5^ mol/L). The samples were mixed for a while using a vortex and incubated in room conditions in a dark place for 30 min. At the end of the incubation, the absorbance of the samples was recorded at 517 nm by a spectrophotometer (Shimadzu UV–vis 1800, Japan). DPPH radical scavenging performance of the samples was expressed as mg TE/100 g by using a calibration curve prepared with different concentrations of Trolox. The measurements were replicated three times with two repetitions.

##### ABTS ^+^ radical scavenging activity

The methodology for the determination of ABTS^+^ radical scavenging activity of both muffin cakes and hydrolysates was followed according to Re et al. (1999). Firstly, an ABTS stock solution was prepared by dissolving ABTS in distilled water to a 7 mM concentration. Then, 2.45 mM of potassium persulfate was used to create ABTS^+^ radical cations by allowing the ABTS solution and persulfate solution to stand in a dark place at room temperature for 16 h. After that, the absorbance was set at 0.7 ± 0.02 at 734 nm by dilution with phosphate buffer. When the reagent was ready, 100 μL of the extract was mixed with 2 mL of ABTS^+^ solution and the absorbance of the samples was measured 6 min later by a spectrophotometer at 734 nm. ABTS^+^ radical scavenging performance of the samples was expressed as mg TE/100 g by using a calibration curve prepared with different concentrations of Trolox. The measurements were replicated three times with two repetitions.

##### Ferric‐reducing antioxidant power (FRAP)

Ferric‐reducing antioxidant power of the samples was determined according to the methods of Benzie and Strain (1996). For this aim, a FRAP solution including 0.3 M acetate buffer (pH = 3.6), 0.01 M TPTZ (2,4,6‐tripyridyl‐s‐triazine) in 0.04 M HCl, and 0.02 M FeCl_3_*6H_2_O as 10:1:1 (v/v/v) was prepared. A 0.075 mL of extract was mixed with 2.25 mL of FRAP solution and 0.225 mL of distilled water. The mixture was vortexed and subjected to incubation for 30 min at 37°C at the dark condition and room temperature. The absorbance of the samples was recorded at 593 nm by a spectrophotometer (UV–vis 1800, Shimadzu, Japan). Ferric‐reducing antioxidant power of the samples was expressed as mg TE/100 g by using a calibration curve prepared with different concentrations of Trolox. The measurements were replicated three times with two repetitions.

#### 2.2.9 Oxidative stability of muffin cake samples

An Oxitest device (Velp Scientifica, Usmate, MB, Italy) was used to determine the oxidative stability of the muffin samples. The samples (20 g) were placed on the equipment and started to give oxygen in accelerated conditions. The pressure and temperature applied were 6 bar and 90°C, respectively. After the test, an induction period was recorded for the samples.

#### 2.2.10 Statistical analysis

For the statistical analysis, SAS 19.0 software (Chicago, IL, USA) was used, and Duncan's test was applied to compare the factors at a confidence level of 0.05 to calculate the rheological parameters, a nonlinear regression analysis was performed using Statistica software. All measurements were replicated with three repetitions.

## 3 RESULTS AND DISCUSSION

### 3.1 Some physicochemical properties of the muffin cake samples

Table 1 shows the basic physicochemical characteristics of the muffin samples enriched with WPI and WPHs at different degrees. As is seen, the moisture, water activity, oil, and protein contents of the samples ranged between 20.86%–21.92%, 0.707–0.721, 13.13%–14.07%, and 10.17%–12.49%, respectively (Table 1). Moisture level was determined as 21.48% for the control samples and the fortified muffins with WPH, it was in the range of 20.68–21.41% and a significant increase was observed for the moisture level with the increase in HD from 5% to 15% (*p* < .01). The water activity also decreased with the addition of WPH in the muffin samplers compared to control. The highest oil content (14.07%) was recorded for the muffin sample enriched with 15% HD while the control muffin sample had 13.99% fat content. The most obvious change was monitored for the protein content of the samples and a significant increase was determined with the addition of isolate and hydrolysates at different degrees. The muffin samples including hydrolysate at 15% HD had a 12.49% protein level while the control sample having no WPI or WPHs had 10.17% protein content (*p* < .05). The values of emulsion stability and also specific volume of the cake batters are tabulated in Table 1. As is seen, emulsion stability values ranged between 10.47% and 77.96%. The highest emulsion stability value was determined for the batter sample added with whey protein hydrolysate at 15% HD while the lowest value was for the control muffin batter sample. It was obviously seen that the increase in the HD increased the stability values of the cake batters significantly (*p* < .05). Ren et al. (2017) investigated the effect of HD on emulsion activity and stability of sunflower protein isolates and reported that the emulsion activity was 33.9 m^2^/g for sunflower protein isolate and it decreased from 24.4 to 14.9 m^2^/g with the increase of HD from 10% to 30%. However, emulsion stability values increased significantly from 25.5% to 76.5% by the increase of HD from 10% to 30% while the emulsion activity of sunflower protein isolate was 24%. It was reported that the excessive hydrolysis process caused a significant loss of emulsion activity because the peptides having low molecular weights could not behave as amphiphilic enough to have the ability to perform good emulsifying properties (Klompong et al., 2007; Taheri et al., 2013). However, for emulsion stability, due to the enzymatic hydrolysis modifying the structural characteristics and causing a decrease in the molecular weight of the peptides, a flexible structure showing good emulsion stability is revealed (McClements, 2007). Similarly, Phongthai et al. (2020) reported that the higher HD for the protein isolates reduced the emulsion activity because the small peptides can migrate easily and have no strong effect on the reduction of the interfacial tension. However, Jamdar et al. (2010) concluded that the HD had a positive effect on the emulsion stability at higher pH values. Specific volume levels of the muffin samples were in the range of 2.43–3.01 g/mL while the muffin added with WPI showed the lowest specific volume and the muffin added with WPHs at 15% HD showed the lower specific volume level compared to the muffin sample added with WPHs at 5% HD. Mohammadi et al. (2022) produced muffins added with lentil protein hydrolysates and reported that the specific volume of the muffin samples ranged between 1.63 and 1.85 g/mL and an increase in the HD in the protein caused a decrease in the specific volume of the muffins. Fitzgerald et al. (2014) reported that the decrease in specific volume with an increase in the degree of hydrolysis of the protein is due to the negative effect of the hydrolysate on the starch‐gelling process. This may be due to the effect of the added protein, which can compete with wheat starch for free moisture, thus restricting the hydration and swelling of starch granules and consequently inhibiting the gelation process.

**TABLE 1:** Some physicochemical properties and color characteristics of the muffin samples.

	Whole cake				Cake batter		Cake crust layer				Cake crumb			
Samples	Moisture (%)	a_w_	Oil (%)	Protein (%)	ES (%)	SV (g/mL)	L*	a*	b*	ΔE*	L*	a*	b*	ΔE*
Control	21.48 ± 0.01^b^	0.712 ± 0.00^b^	13.99 ± 0.01^b^	10.17 ± 0.10^e^	10.47 ± 1.64^e^	2.82 ± 0.01^b^	61.33 ± 1.98^a^	12.89 ± 0.86^b^	32.43 ± 1.66^a^	45.30 ± 0.80^b^	76.16 ± 0.83^a^	−2.14 ± 0.29^a^	25.29 ± 1.03^a^	27.54 ± 1.29^a^
WPI	21.92 ± 0.01^a^	0.721 ± 0.01^a^	13.51 ± 0.02^d^	11.69 ± 0.02^d^	34.40 ± 1.94^d^	2.43 ± 0.02^e^	60.14 ± 0.72^a^	14.02 ± 0.42^a^	32.96 ± 0.39^a^	46.61 ± 0.46^b^	78.48 ± 0.37^a^	−2.43 ± 0.03^a^	25.66 ± 0.32^a^	26.40 ± 0.31^a^
5% HD	20.86 ± 0.01^e^	0.708 ± 0.01^c^	13.60 ± 0.00^c^	12.00 ± 0.29^c^	59.89 ± 0.46^c^	3.01 ± 0.06^a^	59.78 ± 0.68^a^	13.64 ± 0.42^a^	32.41 ± 0.49^a^	46.42 ± 0.51^b^	75.90 ± 0.52^a^	−2.27 ± 0.11^a^	25.33 ± 0.56^a^	27.74 ± 0.54^a^
10% HD	21.17 ± 0.01^d^	0.707 ± 0.00^c^	13.13 ± 0.03^e^	12.13 ± 0.02^b^	69.90 ± 0.72^b^	2.60 ± 0.06^d^	58.64 ± 0.94^a^	13.57 ± 0.38^a^	32.26 ± 0.99^a^	47.18 ± 0.58^b^	76.94 ± 0.99^a^	−2.36 ± 0.13^a^	24.84 ± 0.51^a^	26.72 ± 0.82^a^
15% HD	21.41 ± 0.01^c^	0.707 ± 0.00^c^	14.07 ± 0.05^a^	12.49 ± 0.05^a^	77.96 ± 0.39^a^	2.72 ± 0.03^c^	56.36 ± 1.22^b^	14.24 ± 0.35^a^	32.54 ± 0.92^a^	48.71 ± 0.81^a^	74.32 ± 0.84^b^	−2.16 ± 0.07^a^	25.17 ± 0.55^a^	28.70 ± 0.75^a^

*Note*: The small letters in each column show the significance level for the sample (*p* < .05).

Abbreviations: %HD is the hydrolysis degree of whey protein isolate; 10% HD, Muffin enriched with whey protein hydrolysate (10% HD); 15% HD, Muffin enriched with whey protein hydrolysate (15% HD); 5% HD, Muffin enriched with whey protein hydrolysate (5% HD); Control, Control muffin; ES, Emulsion stability; SV, Specific volume; WPI, Muffin enriched with whey protein isolate.

Color values of the samples determined for both crust layer and crumb are also tabulated in Table 1. L*, a*, and b* values of the crust layers of the muffin samples were in the range of 56.36–61.33, 12,89–14.24, and 32.26–32.96, respectively, while the total color changes ranged between 45.30 and 48.71. As is seen, the incorporation of hydrolyzed whey protein decreased the L* values and increased the a* values with no effect on the b* values. Total color changes in the crust layer of the muffin samples increased significantly with the increase in added whey protein HD. For the color of the crumb of the muffin samples, L*, a*, and b* values ranged between 74.32–78.48, −2.43‐(−)2.16, and 24.84–25.66, respectively, while the total color changes were in the range of 26.40–28.70. As it is known, WPI is used in bakery products such as bread, biscuits, cakes, and crackers due to the golden surface color it provides (Kosikowski, 1979). It has also been reported that WPHs provide much better color properties in different food applications compared to WPI (Jeewanthi et al., 2015). Gani, Broadway, Ahmad, et al. (2015) observed increased ΔE color values in the color properties of cookie samples enriched with milk protein isolates and hydrolysates, similar to our study findings. This can be explained by the increased concentration of proteins interacting with sugars and the occurrence of more nonenzymatic browning reactions. The increase in reactive groups and sites released by hydrolysis treatment may be associated with the fact that hydrolysates exhibit more significant differences in color change compared to isolates (Gani, Broadway, Ahmad, et al., 2015).

### 3.2 Textural properties of the muffin cake samples

Textural characteristics identified as hardness, springiness, cohesiveness, gumminess, and resilience of the muffin samples determined for both crust layer and crumb parts are given in Table 2. As is seen, the crust layer hardness values of the muffin samples ranged between 2833.2 and 4248.2 g while the hardness values were in the range of 3378.4–4758.1 g for the crumb part of the muffin samples. The differences among the hardness values of crust parts of the muffin samples were determined to be significant (*p* < .05). For the crust layer, the addition of hydrolyzed WPI decreased the hardness values of the samples with the increase in HD while the hardest sample was the muffin added with native WPI. However, hardness values increased with the increase in the HD of the WPI while the highest hardness value was recorded for the muffin sample crumb added with native WPI but this change was insignificant (*p* > .01). The results of our study are consistent with the literature results such as Karimi et al. (2021). Springiness values of the crust layer and crumb part of the muffin samples ranged between 0.88–0.89 and 0.87–0.97 N and no significant effect was observed with the addition of hydrolyzed WPI addition in the muffin formulation. Similarly, the cohesiveness values of the muffin samples did not change significantly (*p* > .05) with the addition of the hydrolyzed protein in the muffin formulation, and the cohesiveness values were in the range of 0.60%–0.64% and 0.62%–0.67% for crust layer and crumb part of the muffin samples, respectively. Gumminess values of the muffins ranged between 1703.2–2696.7 g and 2212.6–3184.0 g for the crust layer and crumb part of the muffin samples, respectively.

**TABLE 2:** Texture profile analysis results of both crust layer and crumb part of muffin samples.

	Hardness (g)	Springiness	Cohesiveness	Gumminess (g)	Chewiness (g)	Resilience
Crust layer						
Control	3625.50 ± 533.14^b^	0.88 ± 0.00^a^	0.60 ± 0.02^a^	2173.18 ± 301.08^b^	1915.01 ± 267.92^b^	0.23 ± 0.01^a^
WPI	4248.22 ± 257.66^a^	0.89 ± 0.00^a^	0.64 ± 0.00^a^	2696.67 ± 156.90^a^	2406.50 ± 131.90^a^	0.25 ± 0.01^a^
5%HD	3393.69 ± 171.67^b^	0.89 ± 0.01^a^	0.61 ± 0.01^a^	2053.07 ± 69.11^c^	1816.83 ± 51.11^b^	0.24 ± 0.01^a^
10%HD	3144.87 ± 173.67^b^	0.89 ± 0.00^a^	0.63 ± 0.01^a^	1993.29 ± 130.63^c^	1775.35 ± 117.35^b^	0.25 ± 0.00^a^
15%HD	2833.24 ± 215.84^b^	0.89 ± 0.00^a^	0.60 ± 0.02^a^	1703.23 ± 124.43^d^	1514.69 ± 107.12^c^	0.23 ± 0.01^a^
Crumb						
Control	4659.48 ± 930.67^a^	0.87 ± 0.01^a^	0.62 ± 0.03^a^	2861.71 ± 429.04^a^	2488.09 ± 333.10^a^	0.25 ± 0.02^a^
WPI	4758.07 ± 131.29^a^	0.89 ± 0.01^a^	0.67 ± 0.02^a^	3184.00 ± 197.79^a^	2831.07 ± 210.0^a^	0.29 ± 0.03^a^
5%HD	3776.94 ± 524.21^a^	0.97 ± 0.05^a^	0.67 ± 0.04^a^	2512.75 ± 350.31^a^	2437.19 ± 311.90^a^	0.29 ± 0.02^a^
10%HD	3378.43 ± 479.33^a^	0.90 ± 0.02^a^	0.66 ± 0.01^a^	2212.60 ± 285.06^a^	1981.64 ± 237.05^a^	0.26 ± 0.02^a^
15%HD	4221.72 ± 842.10^a^	0.90 ± 0.05^a^	0.64 ± 0.02^a^	2673.78 ± 474.91^a^	2384.05 ± 372.42^a^	0.26 ± 0.02^a^

*Note*: The small letters in each column show the significance level for the sample (*p* < .05).

Abbreviations: Control, Control muffin; WPI, Muffin enriched with whey protein isolate; 5% HD, Muffin enriched with whey protein hydrolysate (5% HD); 10% HD, Muffin enriched with whey protein hydrolysate (10% HD); 15% HD, Muffin enriched with whey protein hydrolysate (15% HD); % HD is the hydrolysis degree of whey protein isolate.

Gumminess values of the crust layer of muffin samples decreased significantly (*p* < .05) with the increase in the HD; however, it resulted in an insignificant increase in the gumminess values of the crumb part of the muffins while the highest gumminess value for both crust layer and crumb of the muffins was recorded for the sample added with WPI. Similarly, chewiness values of the muffin samples were determined as in the range of 1514.7–2406.5 g and 1981.6–2831.1 g for crust layer and crumb of the muffin samples, respectively. Ammar et al. (2021) reported that the hardness values of the sponge cake fortified with whey protein concentrate increased significantly from 7.31 to 16.92 N. In another research, the incorporation of WPI at high levels caused a more fragile cake with the increment in the hardness values of the sponge cake because of high solubility of whey causing the reduction in the sugar solution occurred the crystallization at high cooking temperatures (Díaz‐Ramírez et al., 2016). de Castro et al. (2017) stated that exposing the WPI to high temperature caused a decrease in the solubility of WPI due to its heat sensitivity and so, because of the rupture in the disulfide bonds caused by heat denaturation, an aggregation causing the solid protein network occurred. Because of all the changes, the cake texture was exposed to significant changes such as a high increase in the hardness values (Ammar et al., 2021; Felfoul et al., 2015).

### 3.3 Antioxidant characteristics of the whey protein hydrolysate and muffin cake samples

**FIGURE 1:**
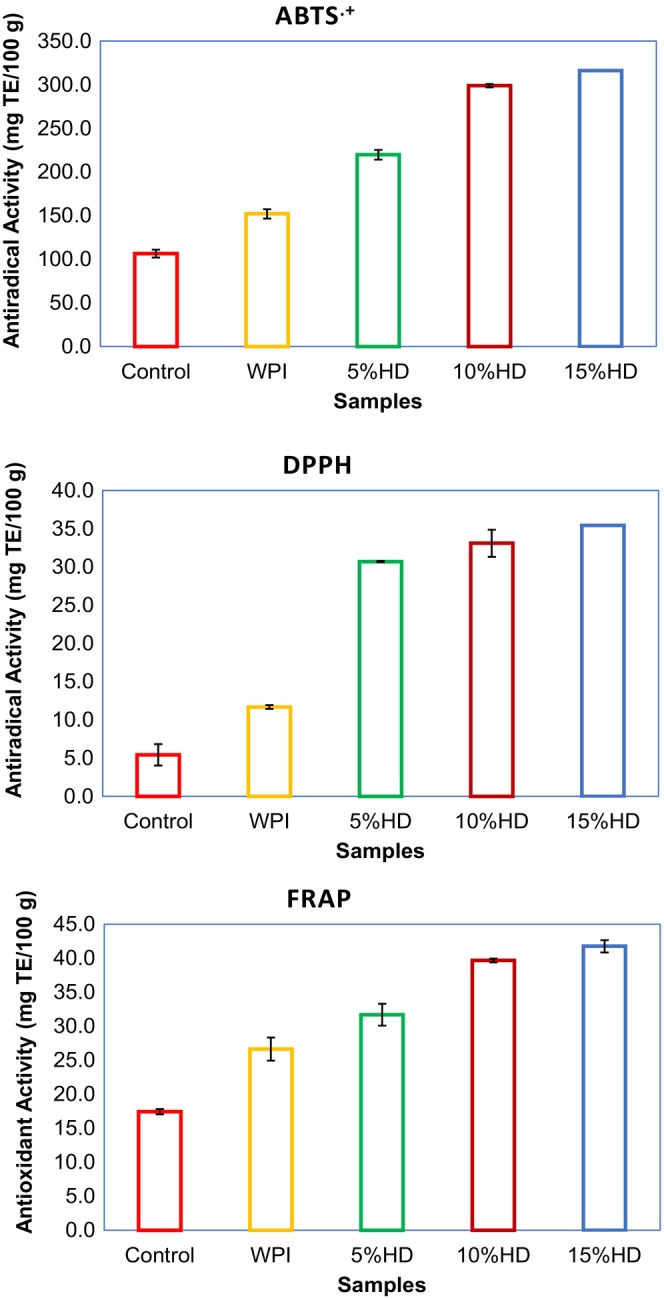
Bioactive characteristics of the muffin samples enriched with whey protein hydrolysates (WPHs). % HD is the hydrolysis degree of whey protein isolate; 10% HD, Muffin enriched with whey protein hydrolysate (10% HD); 15% HD, Muffin enriched with whey protein hydrolysate (15% HD); 5% HD, Muffin enriched with whey protein hydrolysate (5% HD); Control, control muffin; WPI, muffin enriched with whey protein isolate.

Antioxidant and antiradical properties of WPHs and muffin cake samples enriched with WPHs are illustrated in Figure S2 (see supplementary files) and Figure 1, respectively. Ferric‐reducing antioxidant power and ABTS radical scavenging activity ranged between 18.83–87.27 mg TE/100 g and 211.8–5063.1 mg TE/100 g, respectively. The highest FRAP and ABTS values were recorded for the 15% HD WPH while the lowest was for the native WPI. As is seen, an increase in the HD resulted in a significant increase in both antioxidant power and antiradical activity of the WPHs (*p* < .05). Similarly, the antioxidant and antiradical performance of the muffin samples are improved by the addition of the WPHs. DPPH and ABTS radical scavenging activity values were in the range of 7.82–35.43 and 106.4–316.1 mg TE/ 100 g, respectively, while the ferric‐reducing antioxidant power of the muffins was in the range of 17.44–41.77 mg TE/100 g. For all tested bioactive parameters, the control muffin samples showed the lowest values while the muffins had 15% HD WPHs and performed the highest bioactive properties. It was clearly seen that the hydrolyzation process applied to the WPI improved the bioactivity of the final products (Figure 1). You et al. (2009) reported that the DPPH radical scavenging activity of the WPHs increased significantly with the increase in HD. They found that the radical scavenging activity values of the launch WPHs were 83.5 and 95.5 for the 18% HD and 23% HD, respectively. Similarly, Jamdar et al., 2010 reported that the DPPH radical scavenging activity increased from 21% to 51% at a concentration of 2.0 mg/mL with an increase in HD of the peanut protein from 10% to 20%. Al‐Shamsi et al. (2018) studied the effect of hydrolyzation on the ABTS radical scavenging effect of camel milk protein and they reported that the antiradical activity was increased with the hydrolyzation. Similar results for the increased ABTS radical scavenging activity were reported in different research (Bekiroglu et al., 2022; Dryáková et al., 2010). The radical scavenging activity of the WPHs is attributed to the disruption of the native structure of proteins by hydrolysis treatment causing the release of peptides capable of radical binding activity (Liu et al., 2020). Korhonen and Pihlanto (2006) reported that the enzymatic hydrolysis is the most commonly used approach for releasing antioxidant peptides from native parent protein. The exposure of amino acids that were buried in the native conformation of protein, increasing of hydrogen ions availability, and concentration of carboxylic acid groups due to enzymatic hydrolysis also contributes to the augmented antioxidant activity of protein hydrolysates (Lin et al., 2012). Compared to the performances of DPPH and ABTS radical scavenging activity of muffin samples, higher antiradical activity was recorded for the ABTS compared to DPPH. It was explained that the differences between the stereoselectivity of the radicals produced different stoichiometry, and thereby had different interactions with hydrolysates (Zhu et al., 2008). For the effect of hydrolysates on the ferric‐reducing antioxidant power, different results were reported. As to the current results, Khantaphant et al. (2011) reported that the increase in the HD of the protein increased the ferric‐reducing capacity of protein hydrolysates from the muscle of brown stripe red snapper prepared using pyloric caeca and commercial proteases. In another research, alkaline‐solubilized tilapia protein hydrolysate prepared using flavourzyme showed an increase in reducing ferric ion when HD increased (Raghavan & Kristinsson, 2008).

### 3.4 Oxidative stability of the muffin cake samples

**FIGURE 2:**
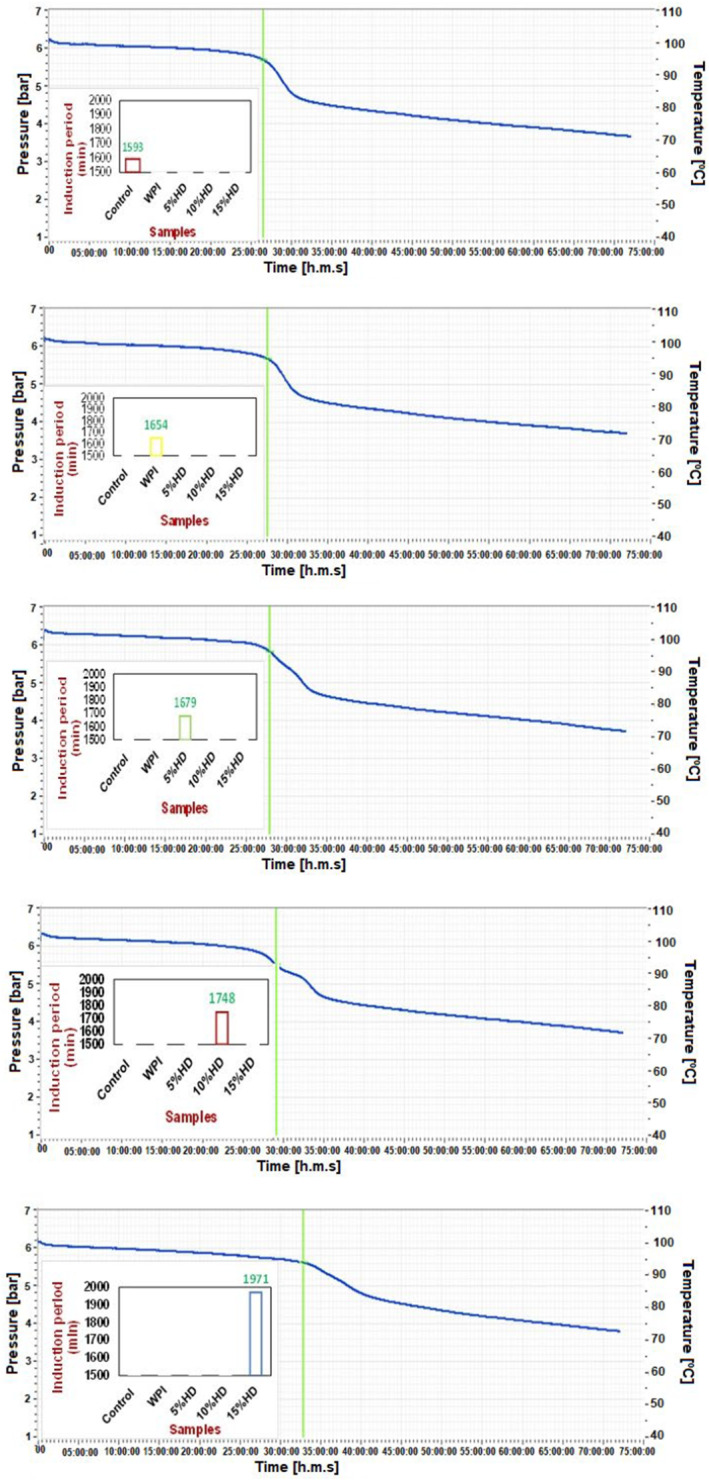
Induction period of the muffin samples enriched with whey protein hydrolysates (WPHs). % HD is the hydrolysis degree of whey protein isolate; 10% HD, Muffin enriched with whey protein hydrolysate (10% HD); 15% HD, Muffin enriched with whey protein hydrolysate (15% HD); 5% HD, Muffin enriched with whey protein hydrolysate (5% HD); Control, control muffin; WPI, muffin enriched with whey protein isolate.

The oxidative stability of the muffin cake samples was characterized by using an induction period which was determined by accelerated oxidation test. The induction period values were calculated from the oxitest graph given in Figure 2. The induction periods were determined as 1593 min for the control muffins, and it was 1654 for the muffin added with native WPI. As is clearly shown from the figures, the induction periods increased significantly with the increase in HD (*p* < .05). The induction period was calculated as 1678 min for the muffin added with 5% HD while the muffins added with 15% HD showed 1971‐min induction period. The increase in the induction periods reveals that the sample showed high antioxidant capacity. In light of this information, a Pearson correlation analysis was performed, and it was observed that significant correlations were determined between induction period and ABTS, DPPH radical scavenging activity, and ferric‐reducing antioxidant capacity with quite high correlation coefficients, namely, 0.846, 0.987, and 0.93, respectively. Wu et al. (2003) reported that a longer induction period signed higher antioxidant performance. They revealed that the increase in the hydrolysis time of the mackerel hydrolysates resulted in an increased induction period showing stronger antioxidant activity. In another research, the higher antioxidant activity of amaranth protein hydrolysate against thermal oxidation of vegetable oil compared to native protein isolate was reported (Tironi & Añón, 2014). Dryáková et al. (2010) reported that the high HD causing high content of released amino groups provided good antioxidant activity. In a study, the inhibition of lipid peroxidation by using WPHs produced by different types of enzyme applications was tested and the researchers reported that all WPHs showed inhibitive activity on lipid peroxidation.

### 3.5 Rheological properties of the muffin batter samples

**FIGURE 3:**
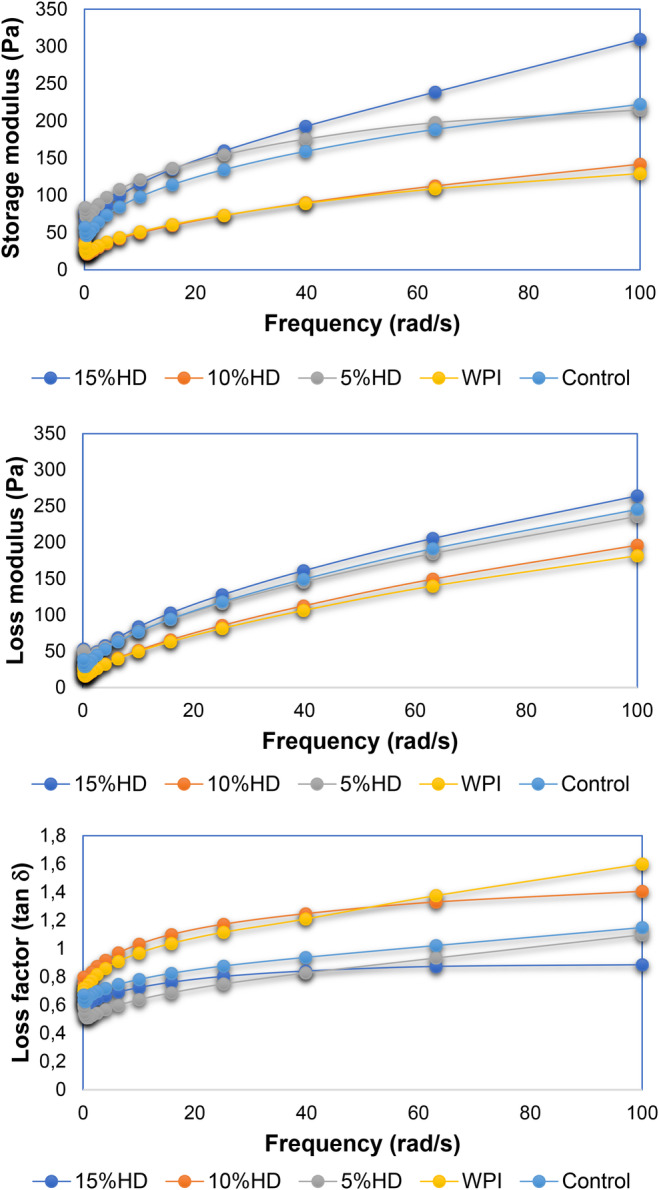
Dynamic mechanical spectra of muffin cake dough enriched with whey protein hydrolysates (WPHs). % HD is the hydrolysis degree of whey protein isolate; 10% HD, Muffin enriched with whey protein hydrolysate (10% HD); 15% HD, Muffin enriched with whey protein hydrolysate (15% HD); 5% HD, Muffin enriched with whey protein hydrolysate (5% HD); Control, control muffin; WPI, muffin enriched with whey protein isolate.

Figure 3 and Table S2 (see supplementary file) illustrate the dynamic shear and steady shear rheograms of the cake batter samples added with WPHs, respectively. As is seen from Figure 3, all batter samples showed viscoelastic behavior and an increase in the frequency resulted in an increase in the recorded dynamic mechanical spectra of the muffin batter samples. For all samples, storage modulus values of the batter samples at constant frequency were determined to be higher storage modulus compared to loss modulus values which showed that the batter sample added with WPHs showed more elastic behavior than the viscous one. In other words, the samples showed a soft gel structure. Matos et al. (2014) reported that the gluten rice‐based gluten‐free muffins enriched with different proteins showed soft gel structure because of the storage modulus (G′) higher than the values of loss modulus (G″) and slight dependence of both moduli with frequency. Similar results were also reported by Marco and Rosell (2008) for the rice flour dough samples showing higher G′ values higher than G″ at the frequency range of 0.1–10 Hz. Elastic and viscous modulus values of the samples were calculated and are tabulated in Table 2. Elastic modulus constant (K′) ranged between 23.94 and 84.85 Pa while the slopes were in the range of 0.20–0.37. The viscous modulus of the samples was in the range of 16.19–36.49 Pa while the slopes were calculated as ranging between 0.42 and 0.54. For the cake batter samples, consistency coefficient values were calculated as in the range of 12.31–29.81 Pa.s^n^ and while the flow behavior index ranged between 0.51 and 0.62. In general, the increase in the WPHs increased the apparent viscosity and consistency of the muffin batter.

### 3.6 Thermal characteristics of the muffin samples

**FIGURE 4:**
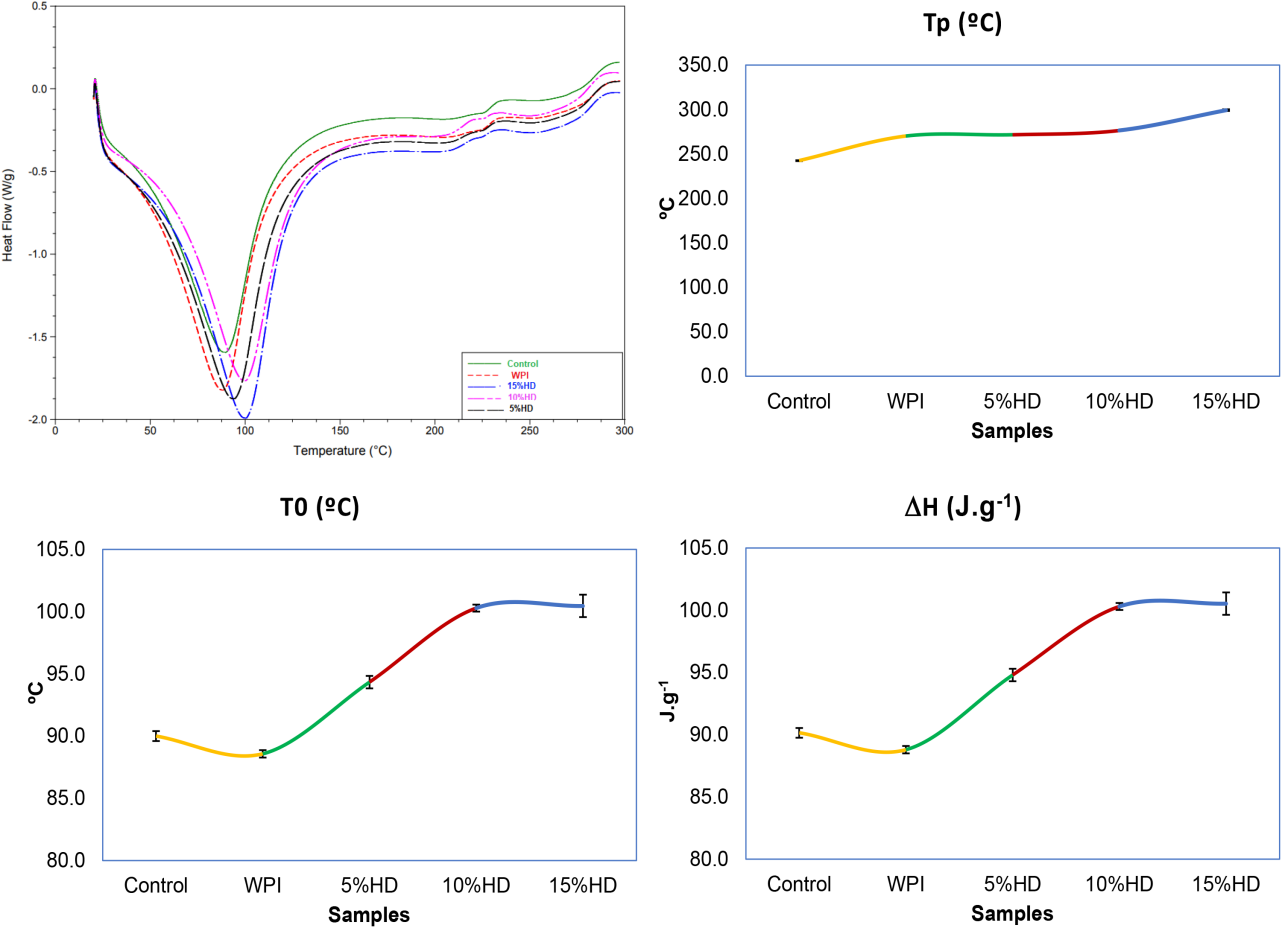
DSC profiles of the muffin samples enriched with whey protein hydrolysates (WPHs). *T*0, Temperature onset, *T*p, Peak temperature, Δ*H*, Gelatinization enthalpy. % HD is the hydrolysis degree of whey protein isolate; 10% HD, Muffin enriched with whey protein hydrolysate (10% HD); 15% HD, Muffin enriched with whey protein hydrolysate (15% HD); 5% HD, Muffin enriched with whey protein hydrolysate (5% HD); Control, control muffin; WPI, muffin enriched with whey protein isolate.

The thermal properties of the baked muffins are given in Figure 4. As is seen, the samples showed similar heat flow behavior, but for the thermal characteristics, muffin samples showed differences. Onset temperature values were recorded as in the range of 88.58–100.46°C while the increase in HD increased the onset temperature values of the samples. Peak temperature values (Tp) of the samples also showed an increment with the increase in HD and the highest peak temperature was recorded for the muffin samples added with WPI having 15% HD, while the lowest peak temperature value was for the control muffin sample. The first endotherm's broadening peak indicated that the protein structure of the substrate was altered by hydrolysis and/or that there are many components, or hydrolysis's byproducts, present that have varying thermal stabilities. In addition, the resulting free groups may react with other components in the muffin, causing stronger bonds to form, resulting in an increase in the temperature and energy required to break these bonds. The transition enthalpy values were determined to be in the range of 88.8–100.5 J/g. It is seen that the addition of hydrolyzed whey protein increased the enthalpy values of the samples. The amount of organized secondary structure in a protein is connected with the value of ΔH, which includes the exothermic and endothermic contributions of the breaking up of hydrophobic and hydrogen bonds, respectively (Privalov, 1974). Furthermore, heat is necessary for the protein to unfold, which increases free volume and molecular mobility. Denaturation and gelatinization are two transitions that entail a process and go to higher temperatures and faster heating rates (Nielsen, 2010). The breakdown of peptide bonds caused by partial enzymatic hydrolysis can change the secondary and tertiary structures of proteins, which can subsequently affect how the modified protein molecule reacts when it is heated to denaturation (Molina Ortiz & Añón, 2001). The substantial intra‐ and intersubunit disulfide linkages provide better stability against thermal denaturation, according to DSC studies of several enzymatic hydrolysates of casein (Farooq, 2019). In a different study, both T_0_ and T_d_ significantly increased with increased hydrolysis time or mean aggregate sizes, while ΔH gradually declined. Compared to the control, WPI hydrolysate (17–625 nm in size) produced a substantially greater ΔH (Ju et al., 1999). Chen and Campanella (2022) reported that the onset and denaturation temperatures of pea protein hydrolysate were higher than native proteins due to the quantity and type of linkages related to the stability of protein aggregation.

## 4 CONCLUSION

Incorporation of WPHs into the muffin cake formulations increased the emulsion stability values of the final cake batter samples and showed a significant effect on some textural parameters such as chewiness while having no effect on hardness and resilience. The muffin cake samples added with WPHs showed high antioxidant and antiradical activity and an increase in HD increased the biofunctional performance of the samples. All batter samples added with hydrolyzed whey protein showed elastic gel character and soft gel structure. Thermal analysis showed that the increase in HD in whey protein increased the transition enthalpy of the muffin cake samples. It was concluded that the WPI could be used as a functional ingredient but to increase the bioactivity of the sample, it should be enzymatically hydrolyzed at a high HD. It is important to note that the choice of protein hydrolysate with a specific HD depends on the intended application and the desired characteristics of the food product.

## AUTHOR CONTRIBUTIONS

**Hatice Bekiroglu:** Conceptualization (equal); data curation (equal); formal analysis (equal); investigation (equal); methodology (equal); validation (equal); writing – original draft (equal); writing – review and editing (equal). **Safa Karaman:** Conceptualization (equal); data curation (equal); methodology (equal); software (equal); supervision (equal); visualization (equal); writing – original draft (equal); writing – review and editing (equal). **Fatih Bozkurt:** Conceptualization (equal); data curation (equal); writing – original draft (equal); writing – review and editing (equal). **Osman Sagdic:** Funding acquisition (equal); project administration (equal); writing – review and editing (equal).

## FUNDING INFORMATION

This research received no external funding.

## CONFLICT OF INTEREST STATEMENT

The authors declare no competing interests.

## ETHICS STATEMENT

This study does not involve any human or animal testing.

## Supporting information


Data S1.

## Data Availability

All data used to prepare this article are included in the tables and figures.
