# Supplementary material for: Characterization of some physicochemical, textural, and antioxidant properties of muffins fortified with hydrolyzed whey protein
Source: Food Sci Nutr. 2024 Aug 20;12(10):8105–17. doi: 10.1002/fsn3.4422 (PMC11521639; doi:10.1002/fsn3.4422)
Supplement: Supplementary file 1 — Data S1. [file FSN3-12-8105-s001.docx]

**Supplementary file**

**A designed fortified muffin by incorporation of hydrolyzed whey protein isolate: Effect of hydrolysis degree on some physicochemical, textural, structural and bioactive properties**

Table S1. Ingredients and their ratios used for the muffin production

| Ingredients (%) | Control | WPI | 5% HD | 10% HD | 15%HD |
| --- | --- | --- | --- | --- | --- |
| Egg | 90 | 90 | 90 | 90 | 90 |
| sugar | 60 | 60 | 60 | 60 | 60 |
| Shortening | 15 | 15 | 15 | 15 | 15 |
| Oil | 15 | 15 | 15 | 15 | 15 |
| Whole fat milk | 60 | 60 | 60 | 60 | 60 |
| Wheat flour | 100 | 95 | 95 | 95 | 95 |
| Baking powder | 4.5 | 4.5 | 4.5 | 4.5 | 4.5 |
| Vanilla | 1 | 1 | 1 | 1 | 1 |
| Whey protein isolate | 0 | 5 | 0 | 0 | 0 |
| Whey protein hydrolysate | 0 | 0 | 5 | 5 | 5 |

Control: Control muffin, WPI: Muffin enriched with whey protein isolate, 5% HD: Muffin enriched with whey protein hydrolysate (5% HD), 10% HD: Muffin enriched with whey protein hydrolysate (10% HD), 15% HD: Muffin enriched with whey protein hydrolysate (15% HD), % HD is the hydrolysis degree of whey protein isolate.

Table S2. Steady and dynamic shear rheological model constants of muffin batters

|  | Steady shear rheological parameters | | | Dynamic shear rheological parameters | | | | | |
| --- | --- | --- | --- | --- | --- | --- | --- | --- | --- |
| Samples | K | n | R^2^ | K′ | n | R^2^ | K′′ | n | R^2^ |
| Control | 15.423 | 0.610 | 0.999 | 57.799 | 0.240 | 0.978 | 29.135 | 0.448 | 0.989 |
| WPI | 12.313 | 0.621 | 0.999 | 24.902 | 0.343 | 0.937 | 16.499 | 0.514 | 0.987 |
| 5%HD | 19.707 | 0.567 | 0.999 | 84.848 | 0.199 | 0.972 | 36.491 | 0.417 | 0.977 |
| 10%HD | 17.638 | 0.582 | 0.999 | 23.937 | 0.372 | 0.959 | 16.186 | 0.536 | 0.986 |
| 15%HD | 29.812 | 0.514 | 0.999 | 61.067 | 0.326 | 0.961 | 35.259 | 0.425 | 0.975 |

Control: Control muffin, WPI: Muffin enriched with whey protein isolate, 5% HD: Muffin enriched with whey protein hydrolysate (5% HD), 10% HD: Muffin enriched with whey protein hydrolysate (10% HD), 15% HD: Muffin enriched with whey protein hydrolysate (15% HD), % HD is the hydrolysis degree of whey protein isolate.

**
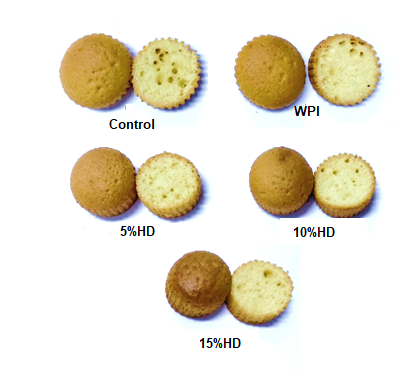
**

Fig. S1 The cake samples enriched with whey protein isolate and hydrolyzates.

Control: Control muffin, WPI: Muffin enriched with whey protein isolate, 5% HD: Muffin enriched with whey protein hydrolysate (5% HD), 10% HD: Muffin enriched with whey protein hydrolysate (10% HD), 15% HD: Muffin enriched with whey protein hydrolysate (15% HD), % HD is the hydrolysis degree of whey protein isolate.

Fig. S2 Bioactive characteristics of whey protein hydrolysates at different degree

WPI: Whey protein isolate, 5% HD: Whey protein hydrolysate with 5% hydrolysis degree, 10% HD: Whey protein hydrolysate with 10% hydrolysis degree, 15% HD: Whey protein hydrolysate with 15% hydrolysis degree,
